# Understanding microRNA Regulation Involved in the Metamorphosis of the Veined Rapa Whelk (*Rapana venosa*)

**DOI:** 10.1534/g3.117.300210

**Published:** 2017-10-27

**Authors:** Hao Song, Lu Qi, Tao Zhang, Hai-yan Wang

**Affiliations:** * Chinese Academy of Sciences Key Laboratory of Marine Ecology and Environmental Sciences, Institute of Oceanology, Chinese Academy of Sciences, Qingdao 266071, China; † Laboratory for Marine Ecology and Environmental Science, Qingdao National Laboratory for Marine Science and Technology, 266071, China; ‡ University of Chinese Academy of Sciences, Beijing 100049, China; § College of Fisheries, Ocean University of China, Qingdao, 266001 China

**Keywords:** miRNA, metamorphic transition, gastropod, larval

## Abstract

The veined rapa whelk (*Rapana venosa*) is widely consumed in China. Nevertheless, it preys on oceanic bivalves, thereby reducing this resource worldwide. Its larval metamorphosis comprises a transition from pelagic to benthic form, which involves considerable physiological and structural changes and has vital roles in its natural populations and commercial breeding. Thus, understanding the endogenous microRNAs (miRNAs) that drive metamorphosis is of great interest. This is the first study to use high-throughput sequencing to examine the alterations in miRNA expression that occur during metamorphosis in a marine gastropod. A total of 195 differentially expressed miRNAs were obtained. Sixty-five of these were expressed during the transition from precompetent to competent larvae. Thirty-three of these were upregulated and the others were downregulated. Another 123 miRNAs were expressed during the transition from competent to postlarvae. Ninety-six of these were upregulated and the remaining 27 were downregulated. The expression of miR-276-y, miR-100-x, miR-183-x, and miR-263-x showed a >100-fold change during development, while the miR-242-x and novel-m0052-3p expression levels changed over 3000-fold. Putative target gene coexpression, gene ontology, and pathway analyses suggest that these miRNAs play important parts in cell proliferation, migration, apoptosis, metabolic regulation, and energy absorption. Twenty miRNAs and their target genes involved in ingestion, digestion, cytoskeleton, cell adhesion, and apoptosis were identified. Nine of them were analyzed with real-time polymerase chain reaction (PCR), which showed an inverse correlation between the miRNAs and their relative expression levels. Our data elucidate the role of miRNAs in *R. venosa* metamorphic transition and serve as a solid basis for further investigations into regulatory mechanisms of gastropod metamorphosis.

Molluscs are biphasic, and metamorphosis is a vital developmental event in their life cycle (Huan *et al.* 2015). In evolutionary terms, animal metamorphosis is crucial because it apparently developed independently in different clades (Hadfield 2000). Competent larvae require a suitable attachment site for metamorphosis, and an unfavorable substrate may be fatal (Bishop *et al.* 2006). Metamorphosis occurs in a relatively short time (generally <48 hr) but is accompanied by high mortality (Huan *et al.* 2015). Therefore, premetamorphosis recruitment and postmetamorphosis survival control mollusc population dynamics (Chandramouli *et al.* 2014). Metamorphosis determines extensive morphological and behavioral changes such as velum degradation and reabsorption, foot proliferation and elongation, and the initiation of rapid secondary shell growth. Thus, elucidating molluscan metamorphic transition attracts considerable theoretical interest among marine biologists.

The rapa whelk, *Rapana venosa*, a mollusc, is widely consumed in China. Efforts involving its commercial aquaculture have been undertaken by many enterprises since 1992 owing to its economic importance (Yuan 1992). However, large-scale aquaculture of this species has been hampered by difficulties with larval culture during metamorphosis. Moreover, wild veined rapa whelk resources have been declining in China owing to increasing fishing activity. This whelk is considered an invasive species beyond the western Pacific Ocean as a result of unintended world-wide transportation, and it heavily threatens the biomass of local bivalves (Yuan 1992). It was first recorded as an invasive species in the Black Sea during the 1940s (Drapkin 1963). Since then, primarily owing to unintended global transport, *R. venosa* has become extremely pervasive and has extended its range to Quiberon Bay, France (Mann *et al.* 2004); Chesapeake Bay (Harding and Mann 1999); Rio de la Plata between Uruguay and Argentina (Pastorino *et al.* 2000); and The Netherlands’ coastal waters (Nieweg *et al.* 2005). Its prevalence heavily disrupts native trophic structure and damages endemic bivalve resources. Metamorphosis may control population dynamics; therefore, understanding its mechanism may be useful for aquaculture, resource restoration, and preventing biological invasion of *R. venosa*.

There are few published reports on *R. venosa* metamorphosis. A previous study described morphological changes that *R. venosa* undergoes in the metamorphosis process; the study also indicated that during this process, the diet of *R. venosa* shifts from phytophagous to carnivorous (Pan *et al.* 2013). Metamorphosis inducers for this species were also investigated, and it was found that acetylcholine chloride and calcium chloride (CaCl_2_) were effective and had low toxicity (Yang *et al.* 2015), suggesting that these compounds may be promising in artificial aquaculture. A comprehensive transcriptome database of *R. venosa* has been constructed from precompetent, competent, and postlarvae (Song *et al.* 2016c), and it forms a baseline for future studies on gene/protein activity associated with metamorphosis. Transcriptomic and proteomic analyses of *R. venosa* metamorphosis identified differentially expressed genes/proteins. This finding indicates that there are multiple processes involved in its metamorphic transition, especially ingestion and digestion, cytoskeleton and cell adhesion, stress response and immunity, and tissue-specific development (Song *et al.* 2016b,d). The metabolic profiles of competent and postlarval stages of *R. venosa* were examined by gas chromatography-mass spectrometry (GC−MS). The analysis detected 53 metabolites whose concentrations differed before and after metamorphosis. They are indicative of the changes in energy metabolism and cell signaling that occur during metamorphosis (Song *et al.* 2016a). Nevertheless, since microRNAs (miRNAs) participate in RNA silencing and post-transcriptional gene expression regulation (Ambros 2004; Bartel 2004), miRNA data are required to provide further concrete support for conclusions drawn from the transcriptome/proteome data.

The miRNAs are short (∼22 nt) and noncoding and have been implicated in cell differentiation, proliferation, migration, and apoptosis. They downregulate the expression of target genes by binding to their 3′ untranslated regions (UTRs) (Sun *et al.* 2017). Metamorphosis is essential for developmental and evolutionary success. Nevertheless, the mechanism by which miRNAs regulate this process remains to be determined. Over the past 10 yr, several studies have been conducted on this aspect in insects, fish, and amphibians. A mutation was found that eliminates let-7 and miR-125 and leads to widespread defects during the metamorphosis of the insects *Blattella germanica* and *Drosophila melanogaster* (Caygill and Johnston 2008; Mercedes *et al.* 2012; Sempere *et al.* 2002). When anti-miR-100 depleted miR-100 in the last instar of the hemimetabolan insect *B. germanica*, the adult wings were slightly smaller than those of the wild type. They also presented with partial fusion of the cross-veins in the anterior remigium and abnormal bifurcations of those in the posterior remigium. The depletion of let-7 elicited the same adult wing vein pattern malformations (Rubio and Belles 2013). The MiR-2 family regulates *B. germanica* metamorphosis by controlling the juvenile hormone signaling pathway (Lozano *et al.* 2015). In the holometabolan *D. melanogaster*, miR-9a-mutants showed wing margin defects and a few ectopic sensory organs (Li *et al.* 2006), and in a later study, miR-9 was found to prevent apoptosis during wing development (Bejarano *et al.* 2010). In the Japanese flounder (*Paralichthys olivaceus*), the expression patterns of 197 miRNAs during metamorphosis were analyzed, and a later study showed that the decrease in miR-17 upregulated Cdc42 during metamorphosis (Zhang *et al.* 2016). In the amphibian *Xenopus tropicalis* and in fish, expression-profiling miRNAs at metamorphosis were identified. The miR-133 played an important part in skeletal muscle development during metamorphosis (Zhang *et al.* 2016). Molluscs include the largest marine phylum, and comprise ∼23% of the total marine organisms. Metamorphosis is the most important developmental event in the molluscan life cycle; however, the characterization and roles of miRNAs in molluscan metamorphosis have not been determined to date.

The purpose of the present study was to elucidate the endogenous miRNAs that drive metamorphosis in the veined rapa whelk *R. venosa*. By sequencing on the Illumina HiSeq 2500 platform, we compared the global expression profiles of small RNAs in precompetent larvae (pre-CL), competent larvae (CL), and postlarvae (PL). In previous studies, we investigated the mRNA global expression profile of whelk metamorphosis (Song *et al.* 2016d); therefore, in the present study we performed a differentially expressed miRNA-mRNA correlation analysis to elucidate miRNA regulation in whelk metamorphosis. These findings will provide new insights into gastropod metamorphosis and facilitate investigation of miRNA function in a biphasic life cycle in the future.

## Materials and Methods

### Sampling

Parent *R. venosa* were collected from their naturally growing areas in Laizhou Bay, China (37°11′4.78″N, 119°41′3.75″E). Parent culture, spawning, larval incubation, and rearing were performed at the Blue Ocean (Laizhou, Shandong, China) according to previously published methods (Pan *et al.* 2013). Planktonic larvae were cultured in 2.5 × 4 × 1.2 m cement pools at 23.5–25.8° and a density of 0.3/ml. *Isochrysis galbana*, *Chlorella vulgaris*, and *Platymonas subcordiformis* were pooled and provided as a daily diet (2 × 10^5^ cells/ml, two times) to the pelagic larvae. Samples from the precompetent larval (three spiral-whorls) stage, the competent larval (four spiral-whorls) stage, and the postlarval (juvenile) stage were collected as three biological replicates, each of which consisted of 40–100 individuals. The samples were inspected under a microscope to ensure that >95% of the individuals were developmentally synchronized. Each replicate was then rinsed with double-distilled H_2_O and flash-frozen in liquid nitrogen until use.

### Library construction and sequencing

Total RNA was extracted from an individual intestine using the RNeasyMini Kit (Qiagen, Germantown, MD) according to the manufacturer’s instructions. The quality and concentration of RNA were measured using a NanoDrop 1000 spectrophotometer (Thermo Fisher Scientific, Waltham, MA). RNA molecules in the size range of 18–30 nt were enriched by polyacrylamide gel electrophoresis (PAGE). The 3′ adapters were added and the 36–44 nt RNAs were enriched. The 5′ adapters were then ligated to the RNAs as well. The ligation products were reverse-transcribed by polymerase chain reaction (PCR) amplification. The 140–160-bp PCR products were enriched to generate a cDNA library and sequenced using Illumina HiSeq 2500 (Gene Denovo Biotechnology, Guangzhou, China).

### Sequence data analysis

Raw data were obtained from base calling on the original image. They were cleaned by removing reads containing poly-N or poly-A/T/C/G and 5′ adapter contaminants, and those missing 3′ adapters or insert tags. Low-quality reads were also eliminated. Other RNAs (tags originating from protein-coding genes, repeat sequences, rRNA, tRNA, snRNA, and snoRNA) were deleted after blasting against the RepeatMasker (www.repeatmasker.org/), the GenBank database (http://blast.ncbi.nlm.nih.gov), and the Rfam database (http://sanger.ac.uk/software/Rfam). No miRNA information for rapa whelk is included in miRBase v. 21.0, so the remaining clean reads were aligned to it with ≤2 mismatches to seek all known precursor/mature miRNAs. The miRNAs with the highest expression for each mature miRNA family were selected as temporary miRNA references. Clean data were aligned to them and their expression levels were calculated by summing the read counts aligned with the temporary miRNA database with ≤2 mismatches. The precursors of the identified miRNAs were predicted. Molecules without a hairpin structure were identified as pseudo-miRNAs. The potentially novel miRNAs were detected by using MIREAP (http://sourceforge.net/projects/mireap/) with stem-loop structure prediction (Chen *et al.* 2013).

By pairwise comparison of the miRNA expressions among precompetent, competent and postlarval samples, the differentially expressed miRNAs were identified and estimated. This procedure conforms to the BGI standard as follows: (1) the expression of the miRNA in each sample was normalized to determine the TPM (transcripts per million). The formula TPM = actual miRNA count/total count of clean reads × 10^6^ was normalized. (2) The final TPM in each larval stage was calculated by averaging three biological replicates. (3) Fold-change and *P*-values were calculated based on the normalized expression as described previously (Sun *et al.* 2017).

### Quantitative real-time PCR

The total RNA isolated as described above was used in real-time PCR. Briefly, 1 μg of total RNA was reverse-transcribed into cDNA using the One Step PrimeScript miRNA cDNA Synthesis Kit (TaKaRa Bio, Kusatsu, Shiga, Japan), following the manufacturer’s directions. For the mRNA quantitative PCR (qPCR) assay, the primers (Supplemental Material, Table S1) were designed using Primer3 (http://primer3.sourceforge.net/releases.php). The reaction proceeded as follows: 95° for 3 min; 95° for 15 sec, 60° for 25 sec, 72° for 15 sec for 40 cycles, and a final 20-min extension step at 72°. RL28 was chosen as a control gene for internal standardization (Song *et al.* 2017). The mRNA expression levels were quantified with the SYBR PrimeScript RT-PCR Kit II (TaKaRa Bio) following the manufacturer’s instructions on an Eppendorf Mastercycler ep realplex platform (Eppendorf, Hamburg, Germany). For the miRNA qPCR assay, the reaction proceeded for 60 min at 37° and 5 sec at 85°. The cDNA was amplified using real-time PCR and platinum SYRB Green qPCR SuperMix-UDG (Invitrogen, Carlsbad, CA) with miRNA-specific forward and reverse primers (Table S1). The 5.8 S rRNA was used as an internal reference gene to normalize the data (Zheng *et al.* 2016). The amplification products were detected by melting curve and gel electrophoresis to ensure primer efficiency and PCR specificity. The relative expression levels of mRNA and miRNA were estimated by the 2^−ΔΔCT^ method. All data were presented as means ± SE (*N* = 3). Statistical significance was analyzed using SPSS v. 18, with *P* < 0.05 being considered significant.

### Target gene prediction and correlation analysis of miRNA-mRNA

The 3′ UTRs from the rapa whelk transcriptome assembly (Song *et al.* 2016c,d) were used as a reference database to predict the target genes with RNA hybrid v. 2.1.2, svm_light v. 6.01, Miranda v. 3.3a, and TargetScan v. 7.0. The targeting criteria were: (1) no mismatch is allowed between 2 and 8 nt on the 5′ end; (2) the G–U matching number cannot be >3; and (3) the minimum free energy (MFE) of the miRNA/target duplex should be >75% of the MFE of the miRNA bound to its perfect complement. The target genes of differentially expressed miRNAs were mapped to the gene ontology (GO) database (http://www.geneontology.org/) and the KEGG (Kyoto Encyclopedia of Genes and Genomes; http://www.genome.jp/kegg/) pathways for GO and KEGG analyses. GO and pathway terms with *P* ≤ 0.05 (Bonferroni’s correction) were considered statistically significant. The differentially expressed mRNA identified in a previous study (Song *et al.* 2016d) and the differentially expressed miRNAs were integrated to analyze the key miRNA-target pairs. Only the inversely correlated miRNA-target pairs with MFE ≤ −18 were screened.

### Data availability

Raw sequencing data were submitted to the GEO (Gene Expression Omnibus) database with accession No. GSE102631. Supplemental materials include the details of primers for qPCR assays (Table S1), the differentially expressed miRNAs during metamorphosis development (Table S2), and miRNA-target pairs of differentially expressed miRNAs (Table S3).

## Results

### MicroRNA library construction

To identify the miRNAs differentially expressed during metamorphosis in rapa whelk, nine small RNA libraries (precompetent larvae: Pre-CL 1, Pre-CL 2, and Pre-CL 3; competent larvae: CL 1, CL 2, and CL 3; postlarvae: PL 1, PL 2, and PL 3) were constructed using Illumina sequencing. A dataset consisting of ∼85,000,000 reads (ranging from 8,235,539 to 10,159,366 among the samples) was obtained after trimming the adapter sequences (Table 1). A BLAST run against the NCBI GenBank, the RepeatMasker, and the Rfam database identified 19,494 (8.75%) to 18,982 (11.77%) unique small RNAs as rRNA, 229 (0.11%) to 635 (0.19%) as snRNA, 28 (0.01%) to 68 (0.03%) as snoRNA, and 1284 (0.62%) to 3619 (1.12%) as tRNA (Table 1). After removing these small RNAs, the remaining RNAs were further analyzed to identify rapa whelk miRNAs against miRBase v. 21.0. A total of 19,878 (12.33%), 20,976 (11.31%), and 18,263 (9.69%) unique known miRNAs were sought in precompetent larvae, 21,033 (9.44%), 17,913 (9.51%), and 16,210 (7.85%) in competent larvae, and 20,573 (6.36%), 24,996 (7.57%), and 22,325 (6.56%) in postlarvae. There were 212 (0.13%), 211 (0.11%), and 209 (0.11%) unique novel miRNAs found in precompetent larvae, 192 (0.09%), 191 (0.10%), and 181 (0.09%) in competent larvae, and 173 (0.05%), 205 (0.06%), and 207 (0.06%) in postlarvae.

**Table 1:** Mapping statistics of the sequencing data

Sample	Total		rRNA	snRNA	snoRNA	tRNA	known_mirna	novel_mirna	transcriptome	unann
Total sRNAs										
Pre-CL 1		8871613 (100.00%)	446583 (5.03%)	3962 (0.04%)	103 (0.00%)	15565 (0.18%)	5976796 (67.37%)	6857 (0.08%)	489213 (5.51%)	1932534 (21.78%)
Pre-CL 2		9550589 (100.00%)	517504 (5.42%)	3535 (0.04%)	91 (0.00%)	16463 (0.17%)	6050397 (63.35%)	8005 (0.08%)	529334 (5.54%)	2425260 (25.39%)
Pre-CL 3		8828363 (100.00%)	430096 (4.87%)	3832 (0.04%)	179 (0.00%)	13317 (0.15%)	5790774 (65.59%)	5642 (0.06%)	548611 (6.21%)	2035912 (23.06%)
CL 1		10694742 (100.00%)	381354 (3.57%)	2904 (0.03%)	293 (0.00%)	9822 (0.09%)	6671240 (62.38%)	5372 (0.05%)	531073 (4.97%)	3092684 (28.92%)
CL 2		10099364 (100.00%)	323604 (3.20%)	3067 (0.03%)	265 (0.00%)	10247 (0.10%)	6615421 (65.50%)	5537 (0.05%)	459241 (4.55%)	2681982 (26.56%)
CL 3		8705674 (100.00%)	336962 (3.87%)	2911 (0.03%)	124 (0.00%)	8560 (0.10%)	5055094 (58.07%)	4126 (0.05%)	515795 (5.92%)	2782102 (31.96%)
PL 1		8235539 (100.00%)	762545 (9.26%)	11571 (0.14%)	394 (0.00%)	79344 (0.96%)	3788195 (46.00%)	3570 (0.04%)	545512 (6.62%)	3044408 (36.97%)
PL 2		9457937 (100.00%)	659629 (6.97%)	13152 (0.14%)	397 (0.00%)	84076 (0.89%)	5051974 (53.42%)	3917 (0.04%)	502551 (5.31%)	3142241 (33.22%)
PL 3		10159366 (100.00%)	683232 (6.73%)	15748 (0.16%)	365 (0.00%)	78210 (0.77%)	5532014 (54.45%)	4606 (0.05%)	541416 (5.33%)	3303775 (32.52%)
Unique sRNAs										
Pre-CL 1		161280 (100.00%)	18982 (11.77%)	260 (0.16%)	30 (0.02%)	1306 (0.81%)	19878 (12.33%)	212 (0.13%)	22477 (13.94%)	98135 (60.85%)
Pre-CL 2		185539 (100.00%)	20339 (10.96%)	269 (0.14%)	31 (0.02%)	1313 (0.71%)	20976 (11.31%)	211 (0.11%)	25368 (13.67%)	117032 (63.08%)
Pre-CL 3		188569 (100.00%)	18343 (9.73%)	249 (0.13%)	50 (0.03%)	1389 (0.74%)	18263 (9.69%)	209 (0.11%)	30383 (16.11%)	119683 (63.47%)
CL 1		222691 (100.00%)	19494 (8.75%)	269 (0.12%)	68 (0.03%)	1497 (0.67%)	21033 (9.44%)	192 0.09%)	21663 (9.73%)	158475 (71.16%)
CL 2		188340 (100.00%)	16852 (8.95%)	220 (0.12%)	59 (0.03%)	1362 (0.72%)	17913 (9.51%)	191 (0.10%)	17988 (9.55%)	133755 (71.02%)
CL 3		206425 (100.00%)	19762 (9.57%)	229 (0.11%)	28 (0.01%)	1284 (0.62%)	16210 (7.85%)	181 (0.09%)	21359 (10.35%)	147372 (71.39%)
PL 1		323650 (100.00%)	32127 (9.93%)	580 (0.18%)	76 (0.02%)	3619 (1.12%)	20573 (6.36%)	173 (0.05%)	27505 (8.50%)	238997 (73.84%)
PL 2		330025 (100.00%)	31100 (9.42%)	635 (0.19%)	62 (0.02%)	3432 (1.04%)	24996 (7.57%)	205 (0.06%)	26410 (8.00%)	243185 (73.69%)
PL 3		340097 (100.00%)	31250 (9.19%)	630 (0.19%)	67 (0.02%)	3424 (1.01%)	22325 (6.56%)	207 (0.06%)	28421 (8.36%)	253773 (74.62%)

### Different expression profiles of miRNAs

**Figure 1:**
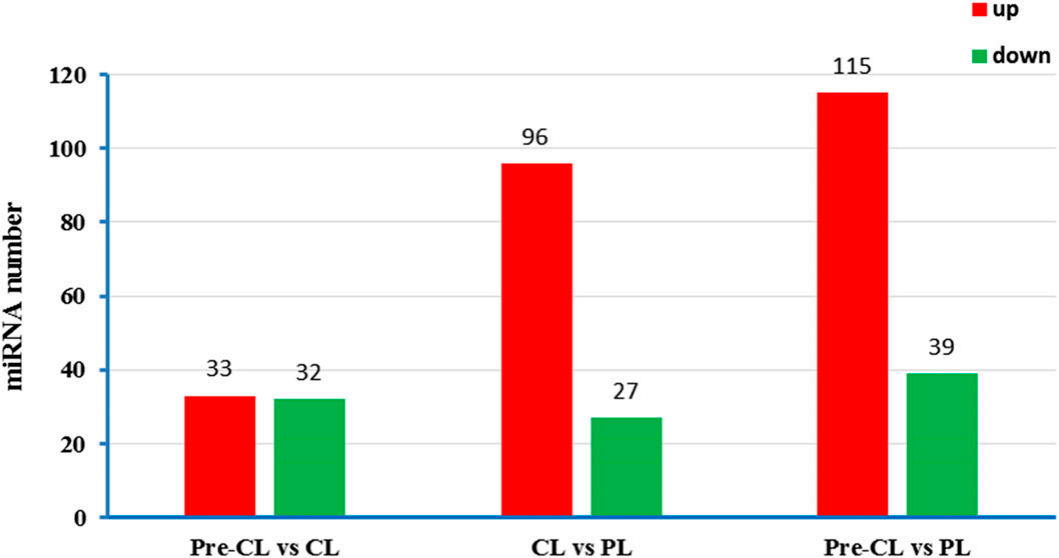
Differentially expressed miRNAs between groups.

The differentially expressed miRNAs were identified by *t*-test with a fold change >2 and *P* < 0.05. A total of 195 miRNAs were obtained, including 33 upregulated and 32 downregulated during the transition from precompetent to competent larvae, and 96 upregulated and 27 downregulated during metamorphosis (Figure 1 and Table S2). Table 2 lists 39 differentially expressed miRNAs with the following criteria: average TPM >10 (in nine samples), log_2_Ratio >2 or <−2 and *P* < 0.01 for ≥1 comparison among the groups. Eleven miRNAs showed a >20-fold difference in gene expression for ≥1 comparison among the groups. These molecules included miR-242-x, novel-m0052-3p, miR-276-y, miR-100-x, miR-183-x, miR-263-x, miR-99-x, miR-37-y, miR-36-y, miR-1175-y, and miR-125-x. These may have important roles in regulating metamorphosis-associated gene expression. Specifically, miR-276-y, miR-100-x, miR-183-x, and miR-263-x showed a >100-fold change, while miR-242-x and novel-m0052-3p expression levels showed a >3000-fold change.

**Table 2:** Thirty-nine differentially expressed miRNAs with the following criteria: average TPM >10 (in nine samples), log2Ratio >2 or <-2 and *P* < 0.01 for ≥1 comparison among groups

miRNA ID	Average TPM			log2_FC (CL/Pre_CL)	*P*-value	log2_FC (PL/CL)	*P*-value	log2_FC (PL/Pre_CL)	*P*-value
	Pre-CL	CL	PL						
miR-242-x	119470.55	63690.16	30.01	−0.91	0.0051	−11.05	0.0001	−11.96	0.0001
novel-m0052-3p	8.17	0.01	36.61	−9.67	0.0001	11.84	0	2.16	0.0222
miR-276-y	44.03	0.16	36.21	−8.14	0.0078	7.85	0.006	−0.28	0.9293
miR-100-x	59.29	23.12	3972.4	−1.36	0.0538	7.42	0.0002	6.07	0
miR-183-x	51432.32	40226.25	406.83	−0.35	0.0222	−6.63	0	−6.98	0
miR-263-x	57623.55	41416.09	513.61	−0.48	0.0218	−6.33	0	−6.81	0
miR-99-x	31.73	9.45	634.7	−1.75	0.0785	6.07	0	4.32	0.0015
miR-37-y	409.54	933.54	23.69	1.19	0.0589	−5.3	0	−4.11	0.0019
miR-36-y	413.61	935.84	24.25	1.18	0.0588	−5.27	0	−4.09	0.0018
miR-1175-y	460.92	435.07	11573.59	−0.08	0.8908	4.73	0.0001	4.65	0.0013
miR-125-x	13.51	5.46	132.68	−1.31	0.5297	4.6	0.0023	3.3	0.0221
miR-92-x	67.63	54.31	3.39	−0.32	0.3449	−4	0	−4.32	0.0001
miR-133-y	52.35	91.6	815.97	0.81	0.1242	3.16	0.0007	3.96	0.0007
novel-m0073-3p	31.68	9.14	2.31	−1.79	0.0016	−1.98	0.0067	−3.77	0.0009
miR-133-z	372.85	582.47	4506.23	0.64	0.2365	2.95	0.0013	3.6	0.0023
miR-278-y	1107.12	2621.61	13402.72	1.24	0.0105	2.35	0.0001	3.6	0.0002
miR-3968-y	35.22	10.92	3.16	−1.69	0.2069	−1.79	0.0032	−3.48	0.023
miR-283-x	489.56	141.62	45.95	−1.79	0.0283	−1.62	0.0004	−3.41	0.0025
miR-1175-x	76.6	45.92	455.17	−0.74	0.3242	3.31	0	2.57	0.0062
miR-124-y	40.89	171.68	388.62	2.07	0.0004	1.18	0.002	3.25	0
miR-1992-y	75.28	45.45	429.55	−0.73	0.6242	3.24	0.0001	2.51	0.0203
miR-9-y	20.04	15.01	132.39	−0.42	0.8649	3.14	0.0003	2.72	0.0162
miR-1986-y	8.86	3.05	25.27	−1.54	0.0618	3.05	0.0004	1.51	0.027
miR-182-x	46.86	21.07	156.48	−1.15	0.3974	2.89	0.0098	1.74	0.0782
novel-m0121-3p	10.19	9.77	68.41	−0.06	0.9767	2.81	0.0007	2.75	0.0036
miR-317-y	115.92	104.18	731.78	−0.15	0.8325	2.81	0.0001	2.66	0.0179
miR-2478-y	1619.2	542.04	262.74	−1.58	0.3887	−1.04	0.0096	−2.62	0.1104
let-7-y	8.32	20.49	46.93	1.3	0.0053	1.2	0.0009	2.5	0.0004
miR-190-x	167.59	226.25	936.13	0.43	0.2449	2.05	0	2.48	0.0032
miR-206-y	57.9	142.27	300.9	1.3	0.0052	1.08	0.0038	2.38	0.0001
miR-216-x	43564.02	25139.85	9272.06	−0.79	0.0012	−1.44	0.0001	−2.23	0.0001
miR-745-y	909.71	285.74	1339.7	−1.67	0.0449	2.23	0.0001	0.56	0.253
miR-281-x	337.71	258.01	1190.95	−0.39	0.0169	2.21	0.0001	1.82	0.0004
miR-278-z	31.68	86.43	141.08	1.45	0.0042	0.71	0.0009	2.15	0.0008
miR-219-x	17.75	7.81	34.07	−1.18	0.4123	2.13	0.0002	0.94	0.1942
miR-277-y	4.52	6.77	19.63	0.58	0.1424	1.54	0.0046	2.12	0.0082
miR-1-z	13128.8	27828.43	54391.53	1.08	0.0017	0.97	0.0016	2.05	0
miR-981-y	3915.01	7628.17	15993.92	0.96	0.0032	1.07	0.0026	2.03	0.0003
miR-278-x	7.45	17.63	30.16	1.24	0.0021	0.77	0.0083	2.02	0.0001

### GO and KEGG pathway enrichment of miRNA target genes

**Figure 2:**
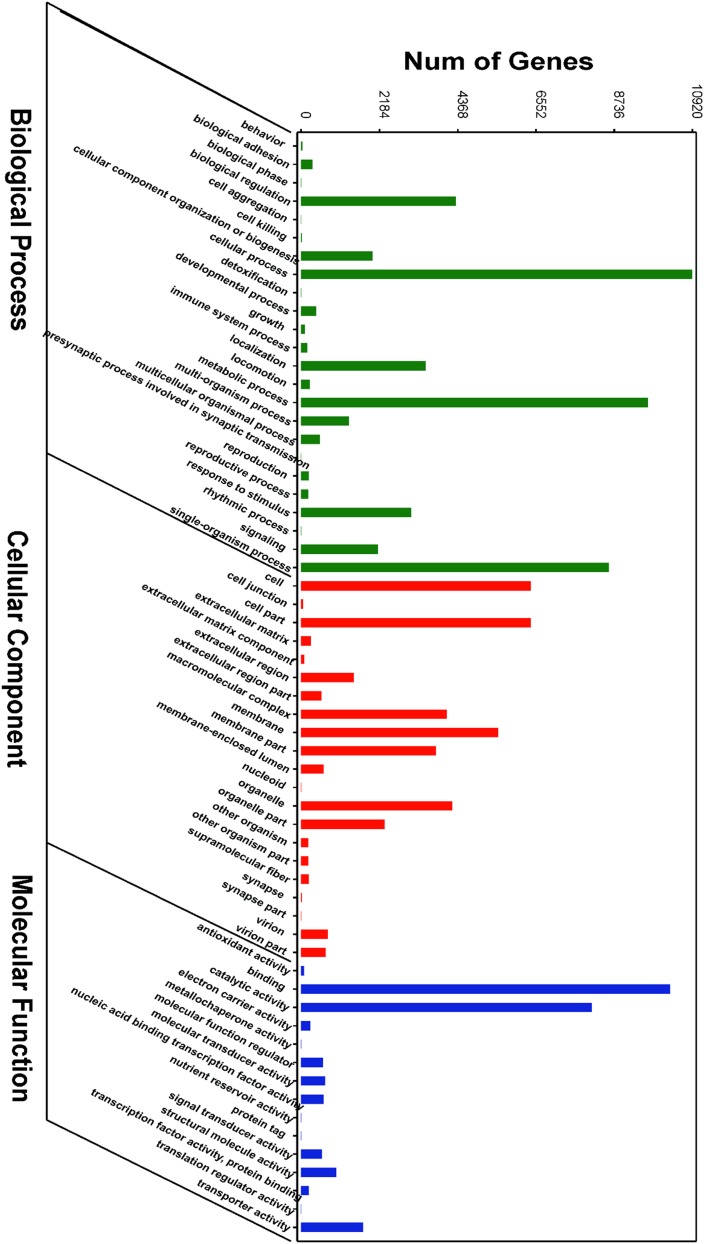
GO terms enrichment of the predicted target genes of differentially expressed miRNAs (*P* < 0.05).

The putative target genes of differentially expressed miRNAs were identified using TargetScan software based on rapa whelk transcriptome libraries (Song *et al.* 2016c). GO analysis was then used to predict enriched functional groups (*P* < 0.05) (Figure 2). “Cellular process (10,917 genes),” “metabolic process (9669 genes),” and “single-organism process (8590 genes)” were the top three enriched items in the biological process, whereas “binding (10,297 genes),” “catalytic activity (8104 genes),” and “transporter activity (1732 genes)” participated in molecular function. “Cell (6406 genes),” “cell part (6406 genes),” “membrane (5487 genes),” “organelle (4219 genes),” and “macromolecular complex (4059 genes)” were highly represented in the cellular component categories.

**Figure 3:**
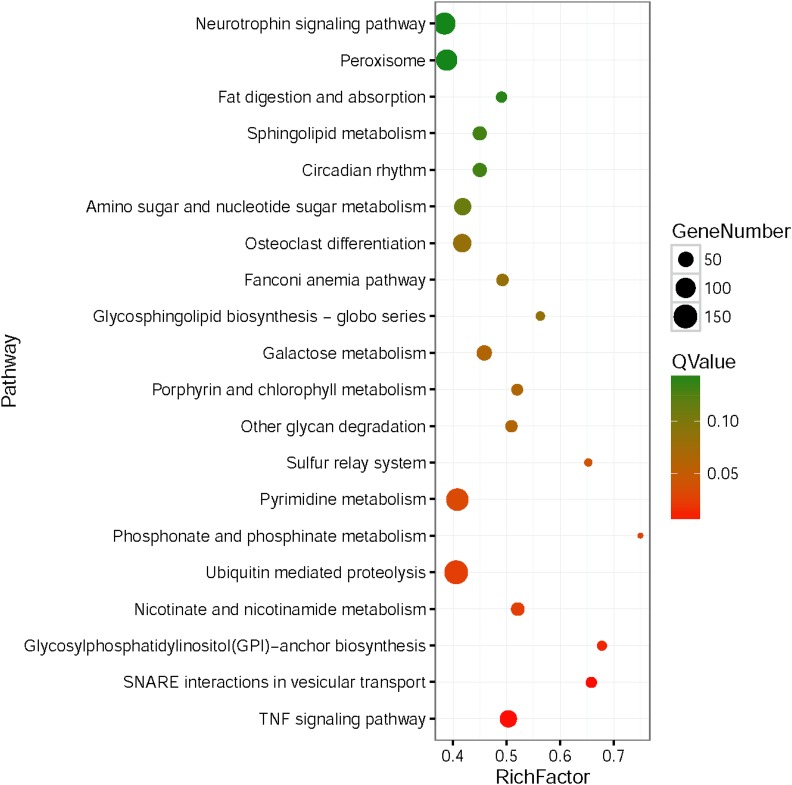
Top 20 pathway enrichment of the predicted target genes of differentially expressed miRNAs. The enrichment factor is the ratio of the number of target genes of differentially expressed miRNAs annotated in this term to the number of all genes annotated in the same term.

Enriched metabolic and signal transduction pathways were identified and are listed in Figure 3. Eight significantly enriched pathways for target genes (Q < 0.05) involved in “TNF signalling pathway,” “SNARE interactions in vesicular transport,” “Glycosylphosphatidylinositol (GPI)-anchor biosynthesis,” “Nicotinate and nicotinamide metabolism,” “Ubiquitin-mediated proteolysis,” “Phosphonate and phosphinate metabolism,” “Pyrimidine metabolism,” and “Sulphur relay system” were screened (Figure 3).

### Selection of miRNA-target pairs and qPCR validation

The aforementioned mRNA expression profiling data from the same metamorphosis sampling stages (Song *et al.* 2016c) were used to perform association analyses along with the current miRNA profiling. The differentially expressed miRNAs detected in the present study were used to select the miRNA-target pairs expressed in metamorphosis (Table S3). The miRNAs negatively regulate the expression of their target mRNAs either by translation inhibition or by mRNA degradation. In previous studies (Song *et al.* 2016b,c), the mRNAs/proteins involved in “ingestion and digestion,” “cytoskeleton and cell adhesion,” and “apoptosis” were thought to have important roles in driving metamorphosis. We identified 20 key miRNA-target pairs potentially implicated in these aspects of whelk metamorphosis (Table 3). For example, in “ingestion and digestion,” we found that let-7-y potentially regulates the SARP-19 precursor, conotoxin Cl14.12, and the exoglucanase XynX genes. Therefore, a single miRNA may regulate multiple target genes during metamorphosis. The miR-1175-x targets the cysteine-rich secretory protein gene, and miR-2001-x targets endo-1,4-β-xylanase. The gene miR-71-x regulates the β-1,4-xylanase and membrane metalloendopeptidase-like 1 genes. In “cytoskeleton and cell adhesion,” tektin-3, dynein heavy chain 8 (axonemal), and dynein intermediate chain 2 (ciliary), all of which are the main structures of velum cilia, were regulated by miR-5106-y, miR-87-y, and miR-315-x, respectively. Novel-m0020-5p determines the expression of apoptosis 2 inhibitor genes, and miR-276-y regulates the caspase-3 gene. These two mRNAs are both important for programmed cell death during metamorphosis.

**Table 3:** Coexpression of 20 key miRNA-targets

No.	miRNA ID	Average miRNA TPM			Target Gene ID	Average mRNA FPKM			Description
		Pre-CL	CL	PL		Pre-CL	CL	PL	
Ingestion and Digestion									
1 *	let-7-y	8.32	20.49	46.93	c112229_g1	252.36	53.19	5.87	SARP-19 precursor
2 *	let-7-y	8.32	20.49	46.93	c124801_g1	9144.35	2226.16	0.13	Conotoxin Cl14.12
3 *	let-7-y	8.32	20.49	46.93	c150903_g1	69.11	2.64	1.59	Exoglucanase XynX
4 *	miR-1175-x	76.60	45.92	455.17	c119967_g1	19.39	22.63	1.05	Cysteine-rich secretory protein
5 *	miR-2001-x	87.40	171.07	278.07	c154241_g2	10.27	1.79	0.02	Endo-1,4-β-xylanase A
6	miR-87-y	1480.69	1819.61	4982.30	c124801_g1	9144.35	2226.16	0.13	Conotoxin Cl14.12
7 *	miR-981-y	3915.01	7628.17	15993.92	c137870_g1	1261.55	85.35	0.10	Endoglucanase E-4
8	miR-71-x	121833.40	97302.93	49293.23	c156029_g2	1.99	4.45	63.30	β-1,4-xylanase
9	miR-67-y	12508.03	20376.51	41820.76	c156902_g1	29.13	6.93	1.45	α-amylase 1
10	miR-71-x	121833.40	97302.93	49293.23	c152193_g1	0.28	15.99	554.55	Membrane metallo-endopeptidase-like 1
Cytoskeleton and Cell Adhesion									
11	miR-5106-y	0.68	0.99	15.98	c155866_g1	833.87	122.39	14.16	Tektin-3
12	miR-87-y	1480.69	1819.61	4982.30	c157287_g2	69.54	17.99	5.11	Dynein heavy chain 8, axonemal
13	miR-315-x	25652.61	30702.55	93369.63	c154991_g1	87.45	11.55	3.14	Dynein intermediate chain 2, ciliary
14	miR-283-x	489.56	141.62	45.95	c137644_g1	1.61	13.81	28.09	Collagen α-5(VI) chain
15 *	miR-263-x	57623.55	41416.09	513.61	c146951_g1	46.76	47.94	82.48	Src substrate cortactin
Apoptosis									
16	novel-m0020-5p	33.74	15.18	10.35	c151900_g1	4.23	4.61	10.52	Apoptosis 2 inhibitor
17 *	miR-276-y	44.03	0.16	36.21	c135194_g1	0.78	2.54	1.25	Caspase-3
Others									
18 *	miR-92-x	67.63	54.31	3.39	c140109_g1	0.00	31.07	50.53	Ependymin
19	miR-183-x	51432.32	40226.25	406.83	c66957_g1	9.40	416.76	1585.15	m7GpppN-mRNA hydrolase
20	miR-216-x	43564.02	25139.85	9272.06	c105989_g1	76.77	128.87	237.52	Cyclin-I

Pairs with asterisks were further analyzed by real-time PCR.

**Figure 4:**
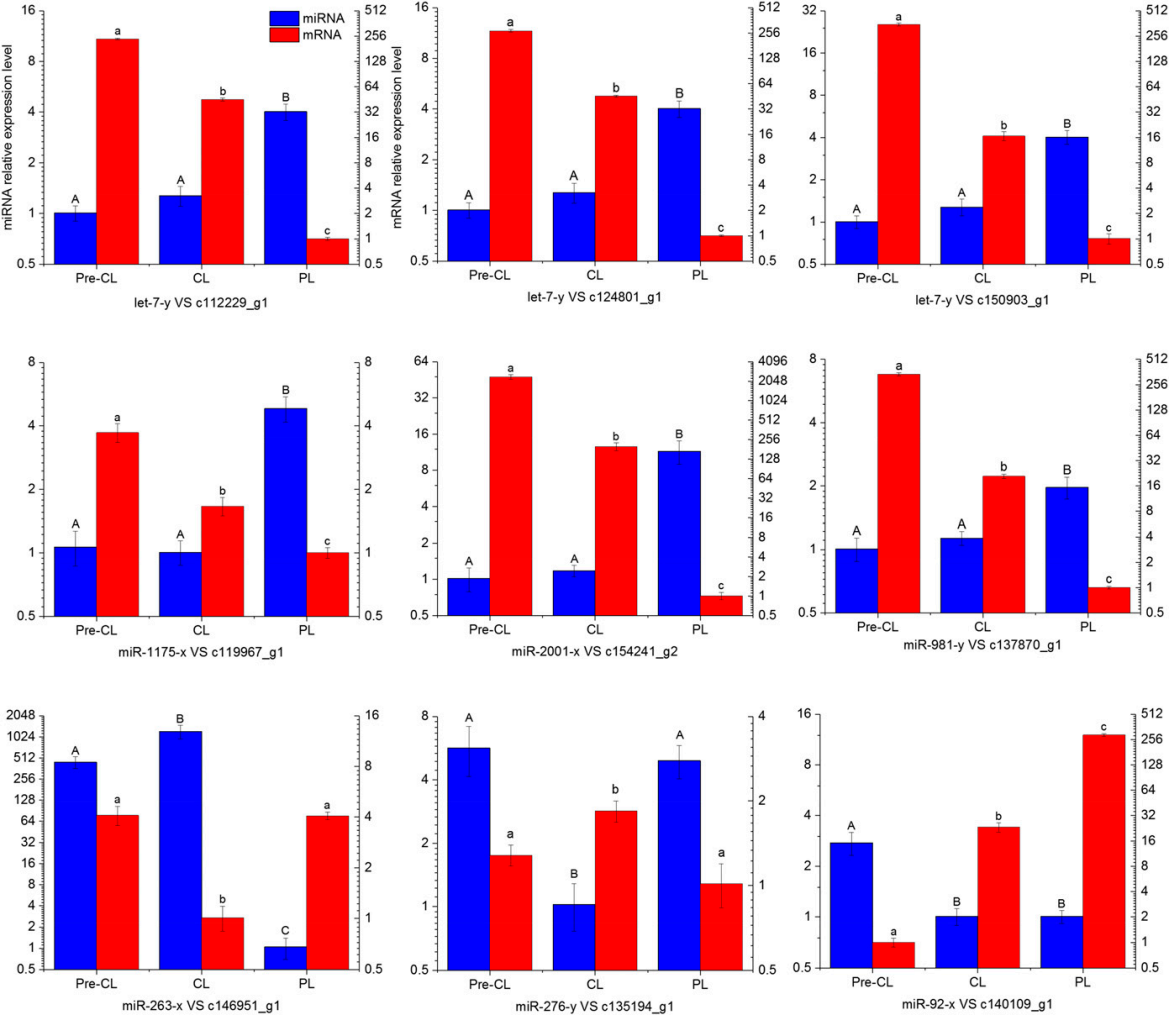
Real-time PCR analysis for nine target miRNA-mRNA pairs. The appearance of the same letter over matching bars means that there is no significant difference between the stages (*P* > 0.05). Different letters over matching bars mean significant differences (*P* < 0.05). Values indicated are means ± SE (*N* = 5).

Real-time PCR analysis was performed on nine key targeted miRNA-mRNA pairs (Figure 4) to validate and identify the metamorphosis-related miRNAs in rapa whelk. The results showed that the miRNAs were consistent with the overall trend in high-throughput sequencing. For each of the nine pairs, there was an inverse correlation between the expression levels of miRNA and miRNA. For example, the let-7-y miRNA decreased during the transition from precompetent to postlarval, whereas its target mRNAs (c112229_g1 SARP-19 precursor, c124801_g1 Conotoxin Cl14.12, and c150903_g1 Exoglucanase XynX) significantly increased.

## Discussion

In this study, we investigated the miRNA expression profile during the metamorphosis of the rapa whelk using high-throughput sequencing. In total, nine libraries were constructed and 85,000,000 reads were obtained. These results will augment information on the small RNA genome of rapa whelk and provide a basis for miRNA regulation during metamorphosis. A total of 195 miRNAs was significantly differentially expressed among three larval stages (precompetent, competent, and postlarval stages) and targeted thousands of genes. This result was expected since metamorphosis is the most complicated developmental event of the life cycle and entails considerable structural, physiological, and behavioral changes.

To obtain an insight into the possible functions of the differentially expressed miRNAs involved in metamorphosis, we performed GO and KEGG pathway enrichment analyses on their predicted target genes. The significantly enriched GO terms “biological adhesion,” “cell aggregation,” “cellular component organisation or biogenesis,” “localisation,” “developmental process,” “signalling,” “immune system,” and “response to stimulus” are primarily associated with development, gene expression, immunity, and the cell cycle. Metamorphosis is a complex process and includes tissue remodeling, cell migration, differentiation, proliferation, and others (Jackson *et al.* 2005). A total of eight significantly enriched pathways were observed. The “TNF signalling” pathways were enriched because they trigger apoptosis, and old organs such as the velum are degenerated by programmed cell death. “SNARE interactions in vesicular transport” were enriched because the nervous system mediates metamorphosis in many marine invertebrates (Voronezhskaya 2004; Gifondorwa and Leise 2006) and SNARE participates in vesicle docking, priming, fusion, and the synchronization of neurotransmitter release into the synaptic cleft during neurosecretion (Shi *et al.* 2011). SNAREs also play a crucial part in the autophagy required for velum degradation and reabsorption during molluscan metamorphosis. “Nicotinate and nicotinamide metabolism,” “Ubiquitin mediated proteolysis,” “Phosphonate and phosphinate metabolism,” “Pyrimidine metabolism,” and “Sulphur relay system” were also affected because of the tissue remodeling and energy redistribution that occur during metamorphosis.

Table 2 lists 39 differentially expressed miRNAs with striking changes. The miR-242-x expression level steadily decreases as precompetent larvae develop into postlarvae. Its expression level decreased by >3000 fold after metamorphosis. Therefore, the miR-242 family may have important roles in rapa whelk metamorphosis. In *Caenorhabditis elegans*, miR-242 and miR-793 target the Argonaute protein ALG-1, which controls the RNA interference process involved in developmental timing (Grishok *et al.* 2001). The expression level of novel-m0052-3p in precompetent larvae remained at an average TPM of 8.17. On the other hand, the average TPM decreased to 0.01 in the competent larvae and rose sharply to 36.61 in the postlarvae. The function of this miRNA remains as yet unknown and, to our knowledge, no relevant studies on it have been published to date. The TPM level of miR-100-x in the precompetent larvae was 59.29. It decreased to 23.12 in the competent larvae but sharply increased to 3974.4 after metamorphosis. There was a similar trend for miR-125-x. Both miR-100 and miR-125 are believed to participate in cell migration. Low levels of miR-100 and miR-125 may promote hepatocellular carcinoma metastasis (Rubio and Belles 2013). Rapa whelk metamorphosis is accompanied by high levels of cell death, proliferation, and tissue remodeling, thus involving the expression of pro-cancer genes, which may regulate cell proliferation and migration. In most insect species, miR-100 clusters with miR-125 in the same primary transcript. These two miRNAs are involved in developmental timing in *C. elegans* and *D. melanogaster* (Rubio and Belles 2013). In the cockroach *B. germanica*, depletion of miR-100 with specific anti-miRNAs in the last instar nymph may reduce adult wing size (Rubio and Belles 2013). In the fruit fly *D. melanogaster*, miR-125 extends the lifespan by repressing *chinmo* in adult brains. Since their concentrations significantly change during rapa whelk development, the functions of these miRNAs in this species are of great interest. In *R. venosa*, miR-9-y was found at a low level (15.01 TPM) in competent larvae but rose to a high level (132.39 TPM) in postlarvae. It may prevent programmed cell death during wing development in *Drosophila* metamorphosis by repressing *Drosophila LIM-only* (Bejarano *et al.* 2010). This function may explain our finding that the miR-9 expression level was low in competent larvae. This developmental stage involves considerable amounts of programmed cell death because the velum must degenerate. Therefore, the levels of miR-9 must be high after metamorphosis in order to suppress further apoptosis.

As stated in previous studies (Song *et al.* 2016b,c), the mRNAs/proteins involved in ingestion and digestion, cytoskeleton and cell adhesion, and apoptosis may also have important roles in driving metamorphosis. Twenty key miRNA-target pairs implicated in these processes were identified, and nine of them were further studied by real-time PCR. Both high-throughput sequencing and real-time PCR showed that the let-7-y expression level continuously increased during metamorphosis, whereas those of its target miRNAs like SARP-19, conotoxin, and exoglucanase continuously declined. The SARP-19 gene was expressed highly in the gastropod larval digestive gland and was sensitive to metamorphic cues (He *et al.* 2014). Conotoxin is a group of neurotoxic peptides isolated from *Conus* venom. High conotoxin levels in *R. venosa* pelagic larvae indicated that this life stage in *R. venosa* is homologous with that of *Conus*. In the rapa whelk, however, it degenerated after metamorphosis (Song *et al.* 2016d). Exoglucanase, an important digestive enzyme in whelk pelagic larvae, sharply decreased when the whelk become carnivorous after metamorphosis. The digestion-related genes were negatively regulated by let-7-y, which implies that let-7 participates in digestive system changes during whelk metamorphosis. The let-7 miRNA also participates in metamorphosis in many other animals. In *Drosophila*, for example, the loss of let-7 and miR-125 may delay the terminal cell-cycle exit in the wing and the maturation of neuromuscular junctions (NMJs) in the adult abdominal muscles. The maturation rate of abdominal NMJs was governed by let-7 during metamorphosis by regulating the expression of the *ab* gene (Caygill and Johnston 2008). In the silkworm *Bombyx mori*, let-7 regulates molting and metamorphosis. Decreased let-7 expression in the silkworm could increase the expression of its target genes *FTZ-F1* and *Eip74EF* (key regulatory factors in the ecdysone pathway) and cause developmental arrest during the larval–larval and larval–pupal transitions (Ling *et al.* 2014). The development-related miR-276 may inhibit apoptosis in shrimp hemocytes (Yang *et al.* 2012). In this study, miR-276 was found to be significantly downregulated in competent larvae, whereas the caspase-3 gene was upregulated. Therefore, miR-276 may regulate apoptosis by targeting the caspase-3 gene. Coexpression studies of key miRNA targets revealed their potential roles in whelk metamorphosis, but the mechanisms by which these miRNAs regulate this developmental process have not been fully explored yet.

In conclusion, the present study provides the first global view of the changes in miRNA that occur during rapa whelk metamorphosis. A total of 195 miRNAs were significantly differentially expressed and their target mRNAs were identified. These molecules are responsible for morphological and functional changes in organs. Some miRNAs involved in ingestion and digestion, cytoskeleton and cell adhesion, and apoptosis during metamorphosis are of great interest and were listed and validated by real-time PCR. These results will provide a basis for understanding the molecular mechanisms involved in the regulation of gastropod metamorphosis.

## Supplementary Material

Supplemental material is available online at www.g3journal.org/lookup/suppl/doi:10.1534/g3.117.300210/-/DC1.
